# The Collection of American Woods at the Central Park Museum

**Published:** 1882-12

**Authors:** 


### THE COLLECTION OF AMERICAN WOODS AT THE CENTRAL PARK MUSEUM.

Among the many curious specimens in the collection now being prepared for exhibition, one which will excite the greatest curiosity is a specimen of the honey locust, which was brought here from Missouri. The bark is covered with a growth of thorns from one to four inches in length, sharp as needles, and growing at irregular intervals. Another strange specimen in the novel collection is a portion of the Yucca tree, an abnormal growth of the lily family. The trunk, about two feet in diameter, is a spongy mass, not susceptible of treatment to which the other specimens are subjected. Its bark is an irregular, stringy, knotted mass, with porcupine-quill-like leaves springing out in place of the limbs that grow from all well-regulated trees. One specimen of the Yucca was sent to the museum two years ago, and though the roots and top of the tree were sawed off, shoots sprang out and a number of the handsome flowers appeared. The tree was supposed to be dead and thoroughly seasoned by this fall, but now, when the workmen are ready to prepare it for exhibition, it has shown new life, new shoots have appeared, and two tufts of green now decorate the otherwise dry and withered log, and the Yucca promises to bloom again before the winter is over. One of the most perfect specimens of the Douglass spruce ever seen is in the collection, and is a decided curiosity. It is a recent arrival from the Rocky Mountains. Its bark, two inches or more in thickness, is perforated with holes reaching to the sap-wood. Many of these contain acorns, or the remains of acorns, which have been stored there by provident woodpeckers, who dug the holes in the bark and there stored their winter supply of food. The oldest specimen in the collection is a section of the *Picea engelmanni*, a species of spruce growing in the Rocky Mountains at a considerable elevation above the sea. The specimen is twenty-four inches in diameter, and the concentric circles show its age to be 410 years. The wood much resembles the black spruce, and is the most valuable of the Rocky Mountain growths. A specimen of the nut pine, whose nuts are used for food by the Indians, is only fifteen inches in diameter, and yet its life lines show its age to be 369 years. The largest specimen yet received is a section of the white ash, which is forty-six inches in diameter and 182 years old. The next largest specimen is a section of the *Platanus occidentalis*, variously known in commerce as the sycamore, button-wood, or plane tree, which is forty-two inches in diameter and only 171 years of age.

Specimens of the red-wood tree of California are now on their way to this city from the Yosemite Valley. One specimen, though a small one, measures five feet in diameter and shows the character of the wood. A specimen of the enormous growths of this tree was not secured because of the impossibility of transportation, and the fact that there would be no room in the museum for the storage of such a specimen, for the diameter of the largest tree of the class is thirty-five feet and eight inches, which represents a circumference of about one hundred and ten feet.

